# Fabrication, Modeling and Characterization of Magnetostrictive Short Fiber Composites

**DOI:** 10.3390/ma13071494

**Published:** 2020-03-25

**Authors:** Zhenjin Wang, Kotaro Mori, Kenya Nakajima, Fumio Narita

**Affiliations:** 1 Department of Materials Processing, Graduate School of Engineering, Tohoku University, Sendai 980-8579, Japan; wang.zhenjin.r8@dc.tohoku.ac.jp (Z.W.); kenya.nakajima.p3@dc.tohoku.ac.jp (K.N.); 2 Department of Mechanical Engineering, Ibaraki University, Nakanarusawa-cho 4-12-1, Hitachi 316-8511, Japan; kotaro.mori.l@vc.ibaraki.ac.jp

**Keywords:** coupled magneto-mechanical modeling, Fe-Co short fiber, flexible composites, inverse magnetostrictive effect, sensors, energy-harvesting

## Abstract

Magnetostrictive materials have a wide variety of applications due to their great capability as sensors and energy-harvesting devices. However, their brittleness inhibits their applications as magnetostrictive devices. Recently, we developed a continuous magnetostrictive Fe-Co-fiber-embedded epoxy matrix composite to increase the flexibility of the material. In this study, we fabricated random magnetostrictive Fe-Co short fiber/epoxy composite sheets. It was found that the discontinuous Fe-Co fiber composite sheet has the magnetostrictive properties along the orientation parallel to the length of the sheet. Finite element computations were also carried out using a coupled magneto-mechanical model, for the representative volume element (RVE) of unidirectional aligned magnetostrictive short fiber composites. A simple model of two-dimensional, randomly oriented, magnetostrictive short fiber composites was then proposed and the effective piezomagnetic coefficient was determined. It was shown that the present model is very accurate yet relatively simple to predict the piezomagnetic coefficient of magnetostrictive short fiber composites. This magnetostrictive composite sheet is expected to be used as a flexible smart material.

## 1. Introduction

Magnetostrictive materials are widely used in many fields as sensors, actuators, energy-harvesting devices, etc. [1]. Among several magnetostrictive materials, Tb-Dy-Fe (Terfenol-D) and Fe-Ga (Galfenol) are widely used for various applications due to their large magnetostriction at room temperature [2,3]. Due to the high cost of Terfenol-D and Galfenol, much effort has been devoted to investigating the magnetostrictive behavior of Fe-Co alloys [4,5], which are lower-cost and have a better workability. Liu et al. [6] designed and fabricated strain-sensitive spin valves with a composite Fe-Ga/Fe-Co layer on a flexible substrate. Bennett et al. [7] demonstrated the doubly clamped resonator design as a magnetoelectric aluminum nitride/Fe-Co thin film heterostructure. Wang et al. [8] also demonstrated the magnetostrictive effect of Fe-Co thin films on a lithium niobate substrate. In order to develop a highly sensitive magnetostrictive material, amorphous Terfenol-D thin films were deposited on a Fe-Co substrate and the influence of heat treatment on the crystal structure, magnetostrictive property, and detection efficiency was studied [9]. On the other hand, magnetostrictive Fe-Co/Fe clad plates [10] and Fe-Co/Ni clad plates [11] were reported to serve as energy-harvesting devices with a high performance. It was shown that the output power of the magnetostrictive clad plates shows a significant improvement in comparison to a single magnetostrictive plate. 

To begin addressing the magnetostrictive effect of the Fe-Co fiber, Narita [12] successfully developed magnetostrictive fiber/polymer composites by embedding Fe-Co fibers, with a diameter of 1 mm in an epoxy matrix and investigated the effect of residual stress on the stress-rate dependent output voltage of the composites due to cyclic compressive loads. Narita and Katabira [13] then discussed, theoretically and experimentally, the output voltage characteristics of the magnetostrictive composites by embedding Fe-Co fibers with a diameter of 0.2 mm in the epoxy matrix. In order to develop Fe-Co-fiber-reinforced polymer composites, the fabric was made by weaving with the warp of polyester fibers and the weft of Fe-Co and polyester fibers and two types of samples were fabricated [14]. It was shown that the magnetic induction change is dependent on the initial magnetization of the Fe-Co fiber and the bias magnetic field. Furthermore, the magnetic induction change of the developed fiber-reinforced polymer composite was comparable to that of bulk Terfenol-D. Recently, Yang et al. [15] fabricated 1–3 metal–matrix Fe-Co/Al alloy composites with a promising capacity in high-temperature applications and examined their microstructure and energy-harvesting properties. Kurita et al. [16] developed magnetostrictive Fe-Co-fiber-integrated shoes and measured the output power and energy of the footstep energy-harvesting during ambulation activities. They showed that the output power and energy depend on the user’s habit of ambulation, not on their weight. In addition, Katabira et al. [17] fabricated the hybrid carbon-fiber-reinforced-polymer (CFRP) composites with Fe-Co fibers and showed that the Fe-Co-fiber-inserted CFRP has a damage self-sensing ability. 

All of the above-mentioned studies have focused on composite materials in which continuous Fe-Co fibers are embedded in the epoxy or aluminum alloy matrix. However, the flexibility of these composites is not good enough to meet the requirement of wearable applications, hence, it is necessary to develop a lighter and more flexible composite material [18,19].

In this investigation, we developed discontinuous Fe-Co fiber/epoxy composite sheets and investigated their magnetic and magnetostrictive properties. The piezomagnetic coefficient of the composite sheets was evaluated. Finite element simulations, coupled with the magneto-mechanical behavior, were also performed to predict the effective piezomagnetic coefficient of the Fe-Co short fiber/epoxy composites. Three-dimensional finite elements were employed to model the representative volume element (RVE) of the composites. On the basis of the averaged magneto-mechanical fields and the definition of the effective magneto-mechanical properties of the composites, the effective piezomagnetic coefficient of the composites with randomly oriented magnetostrictive short fibers was predicted. The numerical predictions were then compared with the test data and the results were examined to give some insights into the magnetostrictive properties of the composites. 

## 2. Experiment

**Figure 1:**
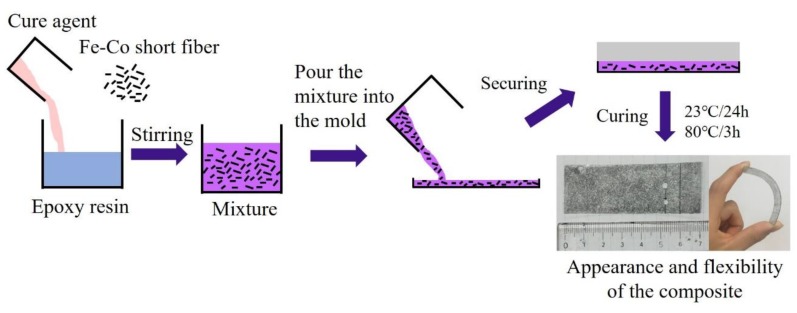
Fabrication process of the discontinuous Fe-Co fiber/epoxy composite.

Figure 1 shows the fabrication process of the discontinuous Fe-Co fiber/epoxy composite sheets. Fe-Co fiber with a diameter of 1 mm was prepared, and wire drawing was successfully executed to obtain a strong textured high-strength fiber with a diameter of approximately 50 μm. The Fe-Co fiber without heat treatment was then chopped to obtain discontinuous Fe-Co fibers with a length of 2 mm. The discontinuous Fe-Co fibers were mixed with an epoxy resin based on diglycidyl ether of Bisphenol-F with a polyamine curing agent. The quantity of Fe-Co fibers was controlled at 2.5 vol.% and 4 vol.%. After pouring and securing, the discontinuous Fe-Co fiber/epoxy precursor was cured for one day at room temperature, and then at 80 °C for 3 h. Figure 1 also shows the appearance and flexibility of the discontinuous Fe-Co fiber/epoxy composite sheet. Fe-Co fibers were homogeneously dispersed in the epoxy resin in a two-dimensional random direction without any agglomeration of Fe-Co fibers inside.

**Figure 2:**
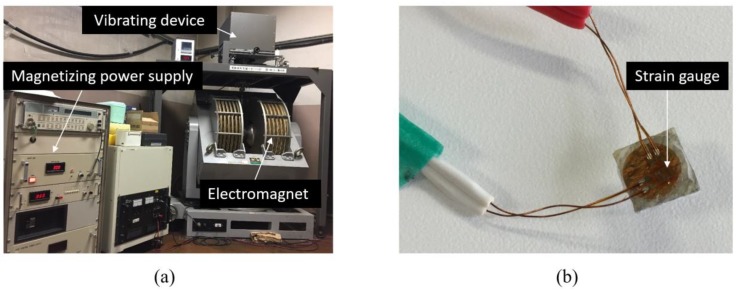
Experimental set up: (**a**) a vibrating sample magnetometer (VSM) machine and (**b**) a magnetostriction measurement specimen.

The fabricated Fe-Co fiber/epoxy composite sheet of length 70 mm, width 25 mm and thickness 0.45 mm was cut to approximately 6 × 6 × 0.45 mm^3^ and their magnetization versus external magnetic field curves (M–H curves) were evaluated by using a vibrating sample magnetometer (VSM), as shown in Figure 2a, with a magnetic field between −7.6 × 10^5^ and 7.6 × 10^5^ A/m in the directions parallel and vertical to the length of the sheet. The strain versus external magnetic field curves were also measured using a strain gauge (see Figure 2b).

## 3. Analysis

### 3.1. Basic Equations

The equilibrium equations for the magnetostrictive materials can be expressed as

(1) $$\sigma_{ji,j}=0$$

(2) $$\begin{array}{cc} e_{ijk}H_{k,j}=0, & \end{array}B_{i,i}=0$$

where *σ_ij_*, *H_i_*, and *B_i_* are the components of the stress tensor, magnetic field intensity vector, and magnetic induction vector, respectively, and *e_ijk_* is the permutation symbol. A comma followed by an index denotes partial differentiation with respect to the space coordinate *x_i_* (*i* = 1, 2, 3). We employed Cartesian tensor notation and the summation convention over repeated indices. Constitutive relations can be given by

(3) $$\varepsilon_{ij}=s_{ijkl}^{H}\sigma_{kl}+d{}^{\prime }{}_{kij}H_{k}$$

(4) $$B_{i}=d{}^{\prime }{}_{ikl}\sigma_{kl}+\mu_{ik}^{}H_{k}$$

where *ε_ij_* is the component of the strain tensor, $s_{ijkl}^{H},d_{kij}^{\prime }$, and *μ_ij_* are the constant magnetic field elastic compliance, magnetoelastic constant, and magnetic permeability, which satisfy the following symmetry equations:

(5) $$s_{ijkl}^{H}=s_{jikl}^{H}=s_{ijlk}^{H}=s_{klij}^{H},\begin{array}{cc} & d{}^{\prime }{}_{kij}=d{}^{\prime }{}_{kji} \end{array},\begin{array}{cc} & \mu_{ij}^{}=\mu_{ji}^{} \end{array}$$

The strain component is given by

(6) $$\varepsilon_{ij}=\frac{1}{2}(u_{j,i}+u_{i,j})$$

where *u_i_* is the component of the displacement vector. The magnetic field intensity vector component is obtained as

(7) $$H_{i}=\varphi_{,i}$$

where *φ* is the magnetic potential. The constitutive Equations (3) and (4) are given by

(8) $$\left\{\begin{array}{c} \varepsilon_{11}\\ \varepsilon_{22}\\ \varepsilon_{33}\\ 2\varepsilon_{23}\\ 2\varepsilon_{31}\\ 2\varepsilon_{12} \end{array}\right\}=\left[\begin{array}{cccccc} s_{11}^{H} & s_{12}^{H} & s_{13}^{H} & 0 & 0 & 0\\ s_{12}^{H} & s_{11}^{H} & s_{13}^{H} & 0 & 0 & 0\\ s_{13}^{H} & s_{13}^{H} & s_{33}^{H} & 0 & 0 & 0\\ 0 & 0 & 0 & s_{44}^{H} & 0 & 0\\ 0 & 0 & 0 & 0 & s_{44}^{H} & 0\\ 0 & 0 & 0 & 0 & 0 & s_{66}^{H} \end{array}\right]\left\{\begin{array}{c} \sigma_{11}\\ \sigma_{22}\\ \sigma_{33}\\ \sigma_{23}\\ \sigma_{31}\\ \sigma_{12} \end{array}\right\}+\left[\begin{array}{ccc} 0 & 0 & d{}^{\prime }{}_{31}\\ 0 & 0 & d{}^{\prime }{}_{31}\\ 0 & 0 & d{}^{\prime }{}_{33}\\ 0 & d{}^{\prime }{}_{15} & 0\\ d{}^{\prime }{}_{15} & 0 & 0\\ 0 & 0 & 0 \end{array}\right]\left\{\begin{array}{c} H_{1}\\ H_{2}\\ H_{3} \end{array}\right\}$$

(9) $$\left\{\begin{array}{c} B_{1}\\ B_{2}\\ B_{3} \end{array}\right\}=\left[\begin{array}{cccccc} 0 & 0 & 0 & 0 & d{}^{\prime }{}_{15} & 0\\ 0 & 0 & 0 & d{}^{\prime }{}_{15} & 0 & 0\\ d{}^{\prime }{}_{31} & d{}^{\prime }{}_{31} & d{}^{\prime }{}_{33} & 0 & 0 & 0 \end{array}\right]\left\{\begin{array}{c} \sigma_{11}\\ \sigma_{22}\\ \sigma_{33}\\ \sigma_{23}\\ \sigma_{31}\\ \sigma_{12} \end{array}\right\}+\left[\begin{array}{ccc} \mu_{11}^{} & 0 & 0\\ 0 & \mu_{11}^{} & 0\\ 0 & 0 & \mu_{33}^{} \end{array}\right]\left\{\begin{array}{c} H_{1}\\ H_{2}\\ H_{3} \end{array}\right\}$$

where

*σ*_23_ = *σ*_32_, *σ*_31_ = *σ*_13_, *σ*_12_ = *σ*_21_ (10)

*ε*_23_ = *ε*_32_, *ε*_31_ = *ε*_13_, *ε*_12_ = *ε*_21_ (11)

(12) $$\begin{array}{l} s_{11}^{H}=s_{1111}^{H}=s_{2222}^{H},\begin{array}{cc} & s_{12}^{H}=s_{1122}^{H}, \end{array}\begin{array}{cc} & s_{13}^{H}=s_{1133}^{H}=s_{2233}^{H}, \end{array}\begin{array}{cc} & s_{33}^{H}=s_{3333}^{H}, \end{array}\\ s_{44}^{H}=4s_{2323}^{H}=4s_{3131}^{H},\begin{array}{cc} & s_{66}^{H}=4s_{1212}^{H}=2\left(s_{11}^{H}-s_{12}^{H}\right) \end{array} \end{array}$$

(13) $$d{}^{\prime }{}_{15}=2d{}^{\prime }{}_{131}=2d{}^{\prime }{}_{223},\begin{array}{cc} & d{}^{\prime }{}_{31}=d{}^{\prime }{}_{311}=d{}^{\prime }{}_{322}, \end{array}\begin{array}{cc} & d{}^{\prime }{}_{33}=d{}^{\prime }{}_{333} \end{array}$$

The constitutive relation of the epoxy matrix is written as:

(14) $$\left\{\begin{array}{c} \varepsilon_{11}\\ \varepsilon_{22}\\ \varepsilon_{33}\\ 2\varepsilon_{23}\\ 2\varepsilon_{31}\\ 2\varepsilon_{12} \end{array}\right\}=\left[\begin{array}{cccccc} \frac{1}{E} & -\frac{\nu }{E} & -\frac{\nu }{E} & 0 & 0 & 0\\ -\frac{\nu }{E} & \frac{1}{E} & -\frac{\nu }{E} & 0 & 0 & 0\\ -\frac{\nu }{E} & -\frac{\nu }{E} & \frac{1}{E} & 0 & 0 & 0\\ 0 & 0 & 0 & \frac{1}{G} & 0 & 0\\ 0 & 0 & 0 & 0 & \frac{1}{G} & 0\\ 0 & 0 & 0 & 0 & 0 & \frac{1}{G} \end{array}\right]\left\{\begin{array}{c} \sigma_{11}\\ \sigma_{22}\\ \sigma_{33}\\ \sigma_{23}\\ \sigma_{31}\\ \sigma_{12} \end{array}\right\}$$

where *E* is the Young’s modulus, *G* is the shear modulus, and *v* is the Poisson’s ratio. The shear modulus *G* is calculated by *G* = *E*/2(1 + *v*). 

### 3.2. Finite Element Model 

**Figure 3:**
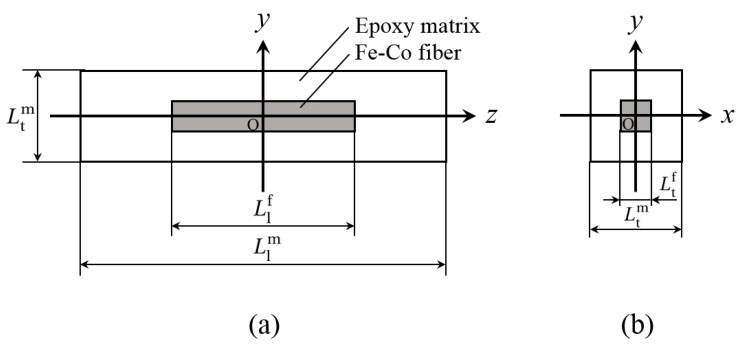
Representative volume element (RVE) model of the discontinuous Fe-Co fiber/epoxy composite: (**a**) *x* = 0 plane and (**b**) *z* = 0 plane.

The RVE of Fe-Co short fiber composites was chosen as a model. Figure 3 shows the RVE model. The Fe-Co short fiber embedded in the matrix was seen as located at the center of the RVE. The local coordinate system (*x*, *y*, *z*) is set up so that the *z*-axis is in the longitudinal direction, and the *x*- and *y*-axes are in the transverse plane of the RVE. In accordance with Figure 3a, $L_{l}^{f}$ represents the length of the Fe-Co short fiber, $L_{t}^{f}$ denotes the length in the transverse direction of the Fe-Co short fiber, and $L_{l}^{m}$ and $L_{t}^{m}$ are the RVE lengths in the longitudinal and transverse directions, respectively. The superscripts f and m denote the Fe-Co short fiber and epoxy matrix. In the RVE model, the Fe-Co fiber size was assumed to keep a constant value and the RVE lengths were changed depending on the volume fraction. The volume fraction *V*^f^ of the Fe-Co short fiber in the RVE can be written in the form

(15) $$V^{f}=\frac{L_{l}^{f}{\left(L_{t}^{f}\right)}^{2}}{L_{l}^{m}{\left(L_{t}^{m}\right)}^{2}},$$

The RVE of the Fe-Co short fiber composites can be assumed to be transversely isotropic, with the plane of isotropy being the *x*–*y* plane as shown in Figure 3b.

**Figure 4:**
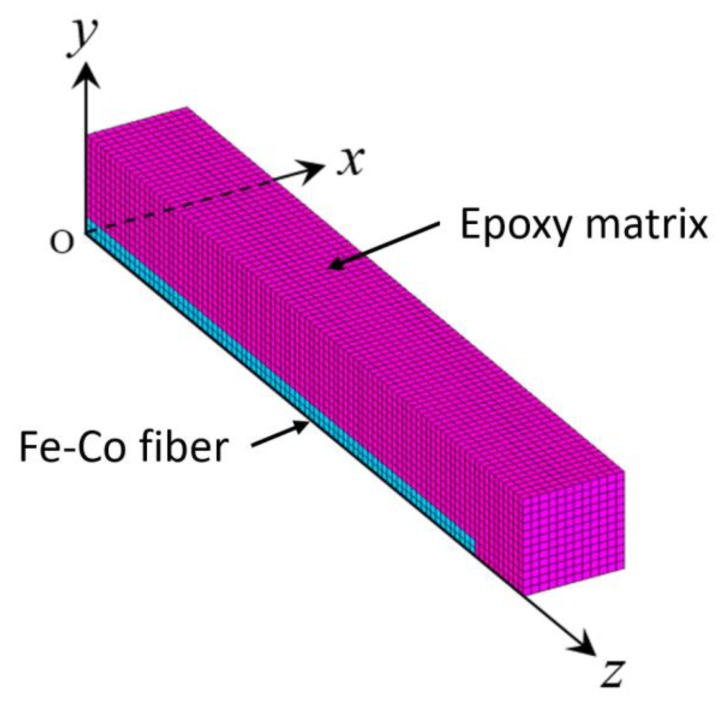
Modeling and meshing of the discontinuous Fe-Co fiber/epoxy composite.

The magnetoelastic constants in Equations (8) and (9) consist of piezomagnetic constants and second-order magnetoelastic constants [20]. Here, we consider only the piezomagnetic constants. The piezomagnetic constants *d*_33_ and *d*_31_ can be obtained from the finite element computations of the RVE under the magnetic field in the *z*-direction. For the magnetic field in the *z*-direction, due to the symmetry, only one-eighth of the RVE (0 ≤ *x* ≤ $L_{t}^{m}$/2, 0 ≤ *y* ≤ $L_{t}^{m}$/2, 0 ≤ *z* ≤ $L_{l}^{m}$/2) is considered (see Figure 4). The displacement boundary conditions for the RVE under a magnetic field in the *z*-direction are

(16) $$u_{x}^{\delta }(0,y,z)=0\begin{array}{cc} & \left(0\leq y\leq L_{t}^{m}/2,0\leq z\leq L_{l}^{m}/2,\delta =f,m\right) \end{array}$$

(17) $$u_{y}^{\delta }(x,0,z)=0\begin{array}{cc} & \left(0\leq x\leq L_{t}^{m}/2,0\leq z\leq L_{l}^{m}/2,\delta =f,m\right) \end{array}$$

(18) $$u_{z}^{\delta }(x,y,0)=0\begin{array}{cc} & \left(0\leq x\leq L_{t}^{m}/2,0\leq y\leq L_{t}^{m}/2,\delta =f,m\right) \end{array}$$

Periodic boundary conditions are

(19) $$\frac{\int_{0}^{L_{l}^{m}/2}\int_{0}^{L_{t}^{m}/2}\sigma_{xx}^{m}(L_{t}^{m}/2,y,z)dydz}{\left(L_{t}^{m}/2)(L_{l}^{m}/2\right)}=0$$

(20) $$\frac{\int_{0}^{L_{l}^{m}/2}\int_{0}^{L_{t}^{m}/2}\sigma_{yy}^{m}(x,L_{t}^{m}/2,z)dxdz}{\left(L_{t}^{m}/2)(L_{l}^{m}/2\right)}=0$$

In this study, the finite element calculation was performed under stress-free conditions on the *z* = $L_{l}^{m}$/2 plane to determine the piezomagnetic constants from the strain generated by the magnetic field. We applied the magnetic induction *B_z_* along the *z*-direction. The magnetic potential is zero on the *z* = 0 plane, and the magnetic field *H_z_*(*x*, *y*, $L_{l}^{f}$/2) is given by the equation, *H_z_* = 2*φ*_0_/$L_{l}^{f}$, where *φ*_0_ is the magnetic potential at *z* = $L_{l}^{f}$/2 plane. The magnetic induction *B_z_*(*x*, *y*, $L_{l}^{f}$/2) is given by the equation, *B_z_* = *μ*_0_*H_z_*, where *μ*_0_ is the magnetic permeability in the epoxy matrix (free space).

The piezomagnetic constants $d_{33}^{\mathrm{uc}}$ and $d_{31}^{\mathrm{uc}}$ of unidirectional aligned Fe-Co short fiber composites are given by

(21) $$d_{33}^{\mathrm{uc}}=\frac{\varepsilon_{zz}^{*}}{H_{z}}$$

(22) $$d_{31}^{\mathrm{uc}}=\frac{\varepsilon_{xx}^{*}}{H_{z}}$$

where ε_zz_^*^ and ε_xx_^*^ are the mean strains in the *z*- and *x*-directions acting on the volume determined from the following conditions

(23) $$\varepsilon_{zz}^{*}=\frac{\int_{0}^{L_{l}^{m}/2}\int_{0}^{L_{t}^{m}/2}\int_{0}^{L_{t}^{m}/2}\varepsilon_{zz}(x,y,z)dxdydz}{(L_{l}^{m}/2){\left(L_{t}^{m}/2\right)}^{2}}$$

(24) $$\varepsilon_{xx}^{*}=\frac{\int_{0}^{L_{l}^{m}/2}\int_{0}^{L_{t}^{m}/2}\int_{0}^{L_{t}^{m}/2}\varepsilon_{xx}(x,y,z)dxdydz}{(L_{l}^{m}/2){\left(L_{t}^{m}/2\right)}^{2}}$$

The finite mesh has 126,144 elements and 137,500 nodes. The mesh of the Fe-Co short fiber has 5000 elements. The model neglects the interaction among the different Fe-Co short fibers. This assumption is not necessarily true, but it does not affect the general conclusions drawn.

The piezomagnetic constants of the unidirectional aligned Fe-Co short fiber composites were predicted through the calculated result of the unit cell. If the composite is a thin plate, a two-dimensional model can be assumed. The effective piezomagnetic constant $d_{33}^{\mathrm{rc}}$ for a two-dimensional randomly orientated Fe-Co short fiber composite is given by

(25) $$d_{33}^{\mathrm{rc}}=\frac{2}{\pi }\int_{0}^{\pi /2}d_{33}^{\mathrm{uc}}\cos\theta d\theta$$

where *θ* is the angle between the orientation of the external magnetic field and the magnetization direction of the Fe-Co short fiber, i.e., the effective piezomagnetic constants are given by multiplying the piezomagnetic constant by the direction cosines.

Table 1 lists the elastic compliances, piezomagnetic constants, and magnetic permeabilities of the Fe-Co bulk. The Young’s modulus and Poisson’s ratio of the epoxy matrix are *E* = 3.78 GPa and *v* = 0.36, respectively.

## 4. Results and Discussion

**Figure 5:**
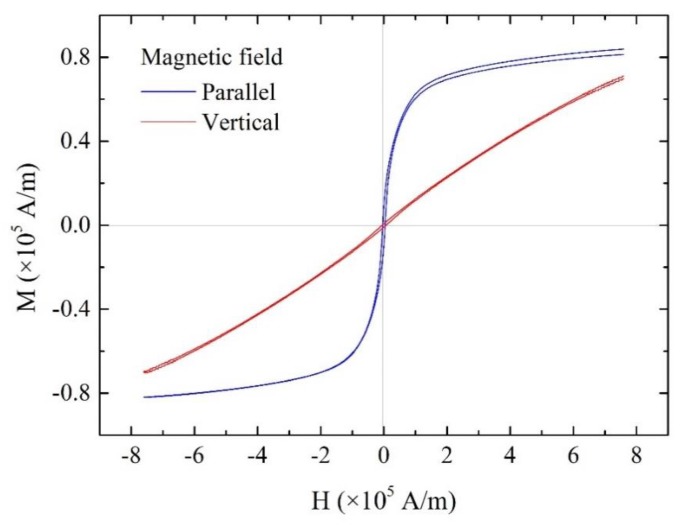
M–H curves of the discontinuous Fe-Co fiber/epoxy composite sheet.

**Figure 6:**
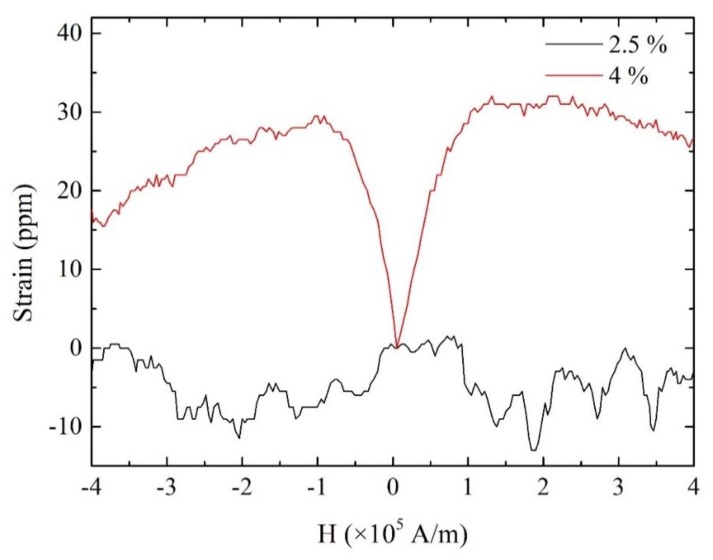
Strain versus external magnetic field of the discontinuous Fe-Co fiber/epoxy composite sheets.

Figure 5 shows the M–H curves of the discontinuous Fe-Co fiber/epoxy composite sheet. Nonlinear behavior was observed in the direction parallel to the length of the sheet, although the behavior was nearly linear in the direction vertical to the length of the sheet. This result indicates that the discontinuous Fe-Co fiber/epoxy composite sheet shows a higher permeability in the direction parallel to the length compared to the vertical direction. The magnetostrictive strain in response to the external magnetic field *H* for the sheets was also examined. Figure 6 shows the strain versus external magnetic field for the discontinuous Fe-Co fiber/epoxy composite sheets with volume fractions of *V*^f^ = 2.5% and 4%. Although the composite sheet with *V*^f^ = 2.5% shows no magnetostrictive behavior, the strain of the composite material with *V*^f^ = 4% increases as the magnetic field increases. It can be seen that the strain saturation is approximately 30 ppm. Moreover, it is interesting to note that the slope (piezomagnetic constant $d_{33}^{\mathrm{rc}}$) is large. 

**Figure 7:**
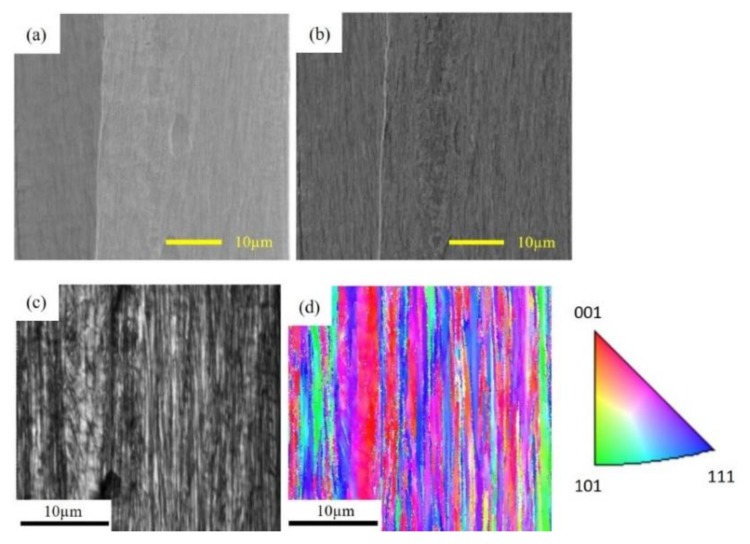
Scanning electron microscope (SEM) image of the Fe-Co fiber with a diameter of 50 μm: (**a**) secondary electron (SE) image, (**b**) back scattered electron (BSE) image, (**c**) image quality (IQ) map and (**d**) inverse pole figure (IPF) map.

In order to predict the effective piezomagnetic constant $d_{33}^{\mathrm{rc}}$, the piezomagnetic constant *d*_33_ of the Fe-Co fiber has to be used because it is expected that the piezomagnetic constants *d*_33_ of the Fe-Co fiber and Fe-Co bulk are different. This is due to the difference of the microstructure between the Fe-Co fiber and Fe-Co bulk. Figure 7 shows the microstructure and crystallographic orientations for the cross-sections of the Fe-Co fiber with a diameter of 50 μm. We found a strong texture in the fiber, that is, the superior texture obtained in the fiber direction due to the wire drawing. It is expected that the value of the piezomagnetic constant *d*_33_ of the Fe-Co fiber will be very high. 

**Figure 8:**
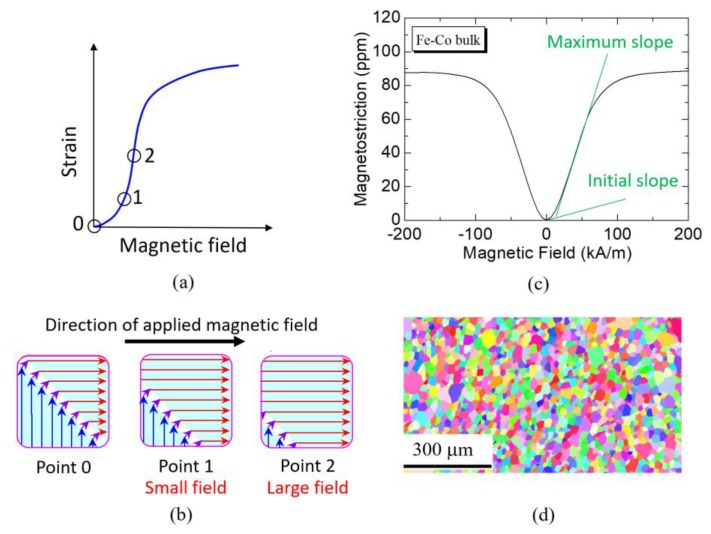
Magnetostriction of the Fe-Co bulk: (**a**,**b**) initial changes and domain movement in the magnetostrictive material, (**c**) magnetostriction curve of the Fe-Co bulk and (**d**) inverse pole figure (IPF) map of the Fe-Co bulk.

The piezomagnetic constant *d*_33_ = 125 × 10^−12^ m/A listed in Table 1 was obtained from the initial slope at the point 0 in the strain versus magnetic field curve, as shown in Figure 8a. Figure 8b shows the domain structures. Applying a larger magnetic field leads to the stronger and more definite re-orientation of more and more domains in the direction of magnetic field. Since it is difficult to measure the piezomagnetic constant *d*_33_ of the Fe-Co fiber, we used the maximum slope at the point 2 in the strain versus magnetic field curve. Figure 8c shows the measured strain versus magnetic field curve of the Fe-Co bulk. There is a strong difference between initial slope and maximum slope. From this figure, a maximum value of *d*_33_ = 1280 × 10^−12^ m/A (maximum slope) was obtained. In order to perform the finite element calculation, we used this value instead of 125 × 10^−12^ m/A (initial slope) as *d*_33_ of the Fe-Co fiber. Figure 8d shows the inverse pole figure (IPF) map of the Fe-Co bulk. Note that the microstructure of the Fe-Co bulk is different from that of the Fe-Co fiber. 

**Figure 9:**
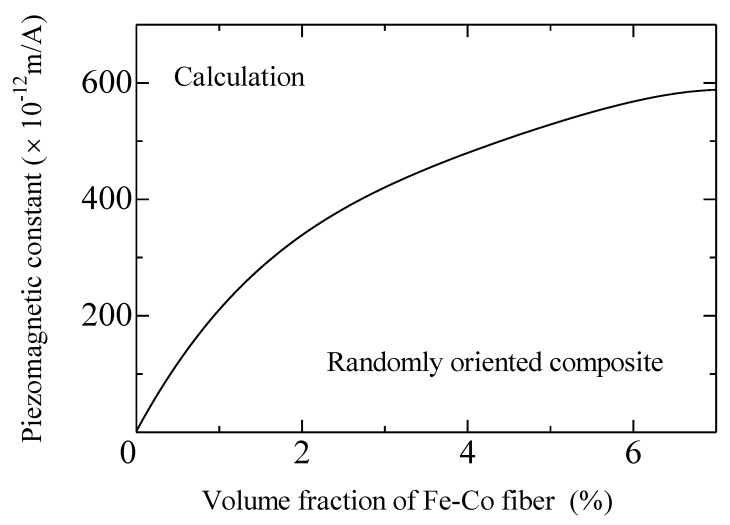
Piezomagnetic constant versus fiber volume fraction of the discontinuous Fe-Co fiber/epoxy composite sheet.

Figure 9 shows the calculated piezomagnetic constant versus volume fraction of the Fe-Co short fiber for the randomly orientated composite. The piezomagnetic constant first increases with an increased volume fraction and then gradually levels off as expected. The value of the composite with *V*^f^ = 4% is approximately 1.25 times higher than the composite with *V*^f^ = 2.5%. However, the experimental results do not show such a tendency (see Figure 6). This may be due to the fact that a smaller volume fraction decreased Young’s modulus of the composite sheet and the strain was not measured correctly. It is well known that gluing the strain gauge on a thin magnetostrictive sample can introduce additional bending stress influencing the real magnetostrictive strain and the magnetostriction measurement using the strain gauge method is difficult for the thin sample [21,22]. The same situation can be applied for soft composite sheets. Table 2 lists the calculated and experimental piezomagnetic constants of the Fe-Co composite with *V*^f^ = 4% and bulk. For the randomly orientated composite, the calculated result is a good match with the experimental result, which indicates that the model can simulate the magnetostriction of the composite correctly. Compared to the Fe-Co bulk, the composites show a larger piezomagnetic constant. The higher piezomagnetic constant demonstrates the higher sensitivity to magnetic field change which is more suitable for energy-harvesting applications. Besides, the calculation results also give us the developed orientation of the Fe-Co-fiber-filled epoxy composites: the unidirectional aligned composite has a 1.6 times higher piezomagnetic constant than the randomly orientated composite. Hence, research regarding the unidirectional aligned composite is needed to increase energy-harvesting efficiency in the future.

## 5. Conclusions

In this study, discontinuous Fe-Co fiber/epoxy composites were successfully fabricated. It was revealed that the discontinuous Fe-Co-fiber-homogeneously-dispersed epoxy matrix composite sheets have magnetostrictive properties along the direction parallel to the length of the composite. 

The Fe-Co fiber/epoxy composite sheet shows a higher permeability in the direction parallel to the length than in the vertical direction. In addition, the magnetostrictive properties shown by the Fe-Co fiber/epoxy composite sheet indicate that the sheet has the potential to be used to harvest energy. 

Furthermore, we succeeded in predicting the piezomagnetic coefficient *d*_33_ of a two-dimensional randomly oriented magnetostrictive short fiber composite sheet with a simple theoretical model. The model can also be used in further simulations of the magnetostrictive composites to increase the efficiency of the development of the composites. The model also gives the future direction of the Fe-Co-fiber-filled composites used for energy-harvesting—a unidirectional aligned fiber-filled composite.

## Figures and Tables

**Table 1:** Material properties of the Fe-Co bulk.

Elastic Compliance (× 10^−12^ m^2^/N)					Piezomagnetic Constant (× 10^−12^ m/A)			Permeability (× 10^−6^ H/m)	
$s_{11}^{H}$	$s_{33}^{H}$	$s_{44}^{H}$	$s_{12}^{H}$	$s_{13}^{H}$	$d_{31}$	$d_{33}$	$d_{15}$	$\mu_{11}$	$\mu_{33}$
5.5	5.5	14.3	−1.65	−1.65	−60.3	125 * 1280 **	318	37.7	37.7

* The value was obtained from the initial slope in the strain versus magnetic field curve. ** The value was obtained from the maximum slope in the strain versus magnetic field curve.

**Table 2:** Calculated and tested piezomagnetic constant of the Fe-Co composite and bulk.

Piezomagnetic Constant (× 10^−12^ m/A)			
-	Unidirectional aligned composite	Randomly oriented composite	Bulk
Calculation Experiment	765 -	484 460	- 125
